# Increased Cytokine Levels Assist in the Diagnosis of Respiratory Bacterial Infections or Concurrent Bacteremia in Patients With Non-Hodgkin’s Lymphoma

**DOI:** 10.3389/fcimb.2022.860526

**Published:** 2022-04-08

**Authors:** Lihua Zhang, Jinping Zhang, Haiping He, Xiaosui Ling, Fan Li, Zefeng Yang, Jinlian Zhao, Huiyuan Li, Tonghua Yang, Shixiang Zhao, Keqian Shi, Xin Guan, Renbin Zhao, Zengzheng Li

**Affiliations:** ^1^ Department of Hematology, The First People’s Hospital of Yunnan Province, Affiliated Hospital of Kunming University of Science and Technology, Kunming, China; ^2^ Yunnan Blood Disease Clinical Medical Center, The First People’s Hospital of Yunnan Province, Kunming, China; ^3^ Yunnan Blood Disease Hospital, The First People’s Hospital of Yunnan Province, Kunming, China; ^4^ National Key Clinical Specialty of Hematology, The First People’s Hospital of Yunnan Province, Kunming, China

**Keywords:** cytokines, IL-6, IL-8, respiratory bacterial infection, bacteremia, non-Hodgkin’s lymphoma (NHL)

## Abstract

Non-Hodgkin’s lymphoma (NHL) is a form of tumor that originates in the lymphoid tissues. Bacterial infections are very common in NHL patients. Because most of the patients do not experience apparent symptoms during the initial stage of infection, it is difficult to detect the underlying condition before it progresses to a more critical level. The activation of the cytokines is a hallmark of inflammation. Due to the advantages of short detection time and high sensitivity of cytokines, many studies have focused on relationship between cytokines and infection. However, few studies have been conducted on NHL patients with infection. Therefore, we reviewed the cytokine profiles of 229 newly diagnosed NHL patients and 40 healthy adults to predict respiratory bacterial infection and bacteremia. Our findings revealed that IL-6(41.67 vs 9.50 pg/mL), IL-8(15.55 vs 6.61 pg/mL), IL-10(8.02 vs 4.52 pg/mL),TNF-β(3.82 vs 2.96 pg/mL), IFN- γ(4.76 vs 2.96 pg/mL), body temperature(37.6 vs 36.5°C), CRP(20.80 vs 4.37 mg/L), and PCT(0.10 vs 0.04 ng/mL) levels were considerably greater in NHL cases with respiratory bacterial infections relative to NHL cases without infection (P<0.05). Furthermore, IL-6(145.00 vs 41.67 pg/mL), IL-8(34.60 vs 15.55 pg/mL),temperature(38.4 vs 37.6°C), PCT(0.79 vs 0.10 ng/mL), and CRP(93.70 vs 20.80 mg/L) levels in respiratory infectious NHL patients with more severe bacteremia were considerably elevated than in patients with respiratory bacterial infections only (P<0.05). Remarkably, increased levels of IL-6 and IL-8 are effective in determining whether or not pulmonary bacterial infectious NHL patients have bacteremia. Temperature, PCT, and CRP all have lower sensitivity and specificity than IL-6. IL-6 ≥18.79pg/mL indicates the presence of pulmonary bacterial infection in newly diagnosed NHL patients, and IL-6 ≥102.6pg/mL may suggest pulmonary bacterial infection with bacteremia. In short, this study shows that cytokines can be advantageous in the diagnosis and differentiation of pulmonary bacterial infection and bacteremia in newly diagnosed NHL patients and may also guide for the use of clinical antibiotics.

## Introduction

Non-Hodgkin’s lymphoma (NHL) is a form of tumor that originates in the lymphoid tissues. NHL patients are at significant risk of infection because of the immunosuppression induced by the malignant tumor and the extreme bone marrow suppression following tumor chemotherapy. Moreover, severe infections are mostly caused by bacteremia, which results in disrupting the therapeutic effects of the treatment and increasing the mortality rate of NHL patients (Jemal et al., 2007; Armitage et al., 2017), and is an independent prognostic criterion in lymphoma patients (Dendle et al., 2017). Infections of the lungs are common in NHL patients. Because most patients do not experience apparent symptoms during the initial stage of infection, it is difficult to detect the underlying condition before it progresses to a more critical level. To reduce the risk of death from serious infections, it is necessary to assess respiratory infections in NHL patients and develop effective early therapies. This emphasizes the importance of biomarkers in the accurate and timely diagnosis of infection and bacteremia.

Inflammatory biomarkers, including C-reactive protein (CRP) and pro-calcitonin (PCT), are routinely utilized for the diagnosis of clinical infection (Shokripour et al., 2020). CRP is an acute-phase reactant that is released in response to infection, tissue injury, and inflammation and is mediated by cytokines, such as interleukin-6, and -1 (Pepys and Baltz, 1983). Multiple factors, including malignancy, anti-inflammatory medicine, and the perioperative phase, can influence CRP levels, making it difficult to evaluate when detected outcomes fluctuate significantly (Nizri et al., 2014; Jukic et al., 2015). PCT is a pro-peptide of calcitonin, which is generally synthesized by the thyroid gland’s C cells. PCT levels in healthy people are very low (less than 0.05 ng/mL), but their levels can be considerably raised in patients with serious septic shock or sepsis (Maruna et al., 2000). Moreover, in cancer patients, the value of PCT may increase due to metastasis, the neuroendocrine function of malignant cells (Whicher et al., 2001), or the production of pro-inflammatory cytokines (MacDonald, 2007). Furthermore, imaging data can be utilized for the diagnosis of bacterial infections; although their sensitivity is limited, specifically in the initial phase of infection. Therefore, patients with malignant tumors need to be combined with other novel markers to make a more accurate diagnosis of infection.

In recent years, cytokines have gained a lot of attention as novel inflammatory indicators that perform a considerable task in the diagnosis of infection. Activated immune cells produce cytokines that enhance the host’s defense against bacterial and viral infections (Akdis et al., 2016). However, abnormal level of cytokines has been linked with the occurrence and severity of several infections. The reported studies revealed that interleukin-6 (IL-6) is a key modulator of the inflammatory response in the initial phase of sepsis, which is closely associated with organ failure (Bian et al., 2017). IL-8 and IL-6 are also abnormally raised in urinary tract infections in non-febrile patients (Rincón-López et al., 2021). Abnormal expressions of IL-6, -8, and -10 have been found in acquired pneumonia (Paats et al., 2013), and significant disruption of cytokines, such as IL-6 and -8 has also been observed in patients infected with COVID-19, which is associated with the severity of infection (Bülow Anderberg et al., 2021). The level of IL-6 can guide management of children with febrile neutropenia and are more specific and sensitive than CRP in the diagnosis of bacteremia/sepsis. IL-6 levels can differentiate gram-positive and gram-negative bacteremia and can predict the survival outcomes (Karan, 2002; Kitanovski et al., 2006; Xu et al., 2013). In addition, cytokines, including IL-6/IL-10/TNF-α/IFN-γ can be used for rapid and timely diagnosis of infection in children with hematological malignancy (Tang et al., 2011). The cytokine pattern of IL-6/IL-10 has more remarkable diagnostic precision in bacteremia and severe infection in children with hematological tumors than PCT (Xia et al., 2016). This evidence suggests that changes in inflammatory cytokines are closely related to infection. In addition, the detection methods of cytokines include real-time quantitative PCR, ELISA, and flow cytometry, among which flow cytometry is increasingly favored for its advantages of high accuracy, small sample size, and fast detection time (i.e., 3-4 hours) (Cook et al., 2001).

The therapeutic value and cost-effectiveness of various treatments should be evaluated as more technologies become available. Cytokines have previously been demonstrated to predict lung bacterial infection in recently diagnosed adult hematological malignancies (Li et al., 2021). In order to evaluate the predictive impact of cytokines for respiratory bacterial infection and respiratory infection associated with bacteremia in NHL, we examined the body temperature, cytokine profile, PCT, and CRP of 229 newly diagnosed NHL patients.

## Material and Methods

### Patient Characteristics and Diagnostic as Well as Inclusion Criteria

We examined a retrospective analysis of 40 healthy people and 229 adults newly diagnosed NHL patients who did not receive any treatment (such as antibiotics and chemotherapy) from January 1, 2018, to January 1, 2021, at the Department of Hematology, the First People’s Hospital of Yunnan Province. CRP, PCT, and cytokines were also measured in 40 healthy individuals. The age of all contributors was ≥ 18 years, 76 of the 229 cases had a confirmed respiratory bacterial infection alone, 15 patients diagnosed respiratory bacterial infection with bacteremia, and the remaining 138 patients were free of infection. Before therapy, all patients’ PCT, CRP, Th1/Th2/Th17 cytokine profiles, and body temperature (axillary temperature) were recorded. This study included only NHL patients with bacterial lung infection or bacterial lung infection in combination with bacteremia. Patients who were infected with viruses, fungi, or other pathogens were excluded from the study.

Refer to the following diagnostic criteria for bacterial lung infection (Lim et al., 2009; Metlay et al., 2019): Regardless of the presence or absence of bacterial cells in respiratory specimens, suspected or confirmed bacterial lung infections were characterized according to clinical and laboratory guidelines. Clinical criteria included a worsening or new cough, shortness of breath, hypoxia, or dyspnea. Additional adjunct examinations included chest X-rays to reveal inflammatory changes and sputum cultures to find infections. A chest CT scan was conducted to rule out respiratory infection in some patients with a suspected lung infection. Bacteremia is described as a positive blood culture that is accompanied by infection-related clinical symptoms (Chirouze et al., 2002; Wiggers et al., 2016).When coagulase-negative staphylococcus, corynebacterium, or bacillus are found in single blood culture, the culture is regarded as contaminated or indicates transient bacteremia, but not an episode of bacteremia. Patients who have a single positive blood culture and those who do not have a positive blood culture are classified as having a non-bacteremia episode. The research was executed following the Declaration of Helsinki, and the Ethics Committee of Yunnan Province’s First People’s Hospital gave its approval. All of the patients gave their informed consent.

### Evaluation of Cytokine Level

The AimPlex bead-based assay (QuantoBio, Tianjin, China) was utilized to evaluate serum cytokine levels (Th1/Th2/Th17), comprising IL-1β, -2, -4, -5, -6, -8, -10, -12P70, -17A, -17F, -22, tumor necrosis factor (TNF) -α and -β, as well as interferon-γ (IFN-γ), and the detection limit was 1 to 2500 pg/mL.

### Statistical Evaluations

For data description, descriptive statistics were used. Median and Inter Quartile Range (IQR) were employed for the representation of continuous data, whereas percentages and frequencies were used to explain categorical variables. A normality test was performed on the data to determine whether it was distributed normally. The Student’s T-test and the Mann-Whitney U test were then implemented for the evaluation of mean variations between two groups with normal and non-normal distributions, accordingly. The Spearman test was used to analyze the correlation between continuous variables and categorical variables. We used a ROC curve to compute the area under the curve (AUC) in order to appraise the diagnostic efficacy of PCT, CRP, and cytokines. The highest Youden index (sensitivity + specificity – 1) was used to find the optimum cut-off value. The distribution map was created using GraphPad Prism 7 and IBM SPSS 21.0 (Armonk, NY, USA) was implemented for statistical assessments.P-value <0.05 (two-sided) was considered statistically meaningful.

## Results

### Patients’ Demographics and Clinical Characteristics

The basic clinical features of patients are given in **Table 1**. The analysis included 229 NHL patients, including 130 males (56.7%) and 99 females (43.3%), with a mean age of 53 years (range 37-62 years). B-cell NHL was found in approximately 71.2% (163/229) of patients, while T-cell and NK-cell lymphoma were found in 19.2% (44/229) and 9.6% (22/229) of patients, respectively. About 74.7% (171/229) of patients had tumors that were stage III or higher. The average body temperature of 43.2% (99/229) of patients was 37.3°C. About 91 patients had bacterial infections (76 had a respiratory infection only, and 15 had a respiratory infection with bacteremia), while the remaining 138 patients had no infection.

**Table 1:** Clinical characteristics of all patients (n=229).

Characteristics		No. of episodes (%)
Age [years, mean (range)]	—	53 (37-62)
Gender	Male	130 (56.7%)
	Female	99 (43.3%)
IPI (scores)	0-2	113 (49.3%)
	3-5	116 (50.7%)
ECOG (scores)	0-2	132 (57.6%)
	3-5	97 (42.4%)
Tumor Stage	I-II	58 (25.3%)
	III-IV	171 (74.7%)
Classification of NHL	T	44 (19.2%)
	B	163 (71.2%)
	NK/T	22 (9.6%)
Chemotherapy	CHOP ± rituximab	150 (65.5%)
	Others	79 (34.5%)
Temperature (°C)	<37.3	130 (56.8%)
	≥37.3	99 (43.2%)

NK, natural killer; CHOP, cyclophosphamide, doxorubicin, vincristine, prednisone.

### Comparison of Serum CRP, PCT, and Cytokine Levels of NHL Patients With or Without Bacterial Respiratory Infection

**Figure 1:**
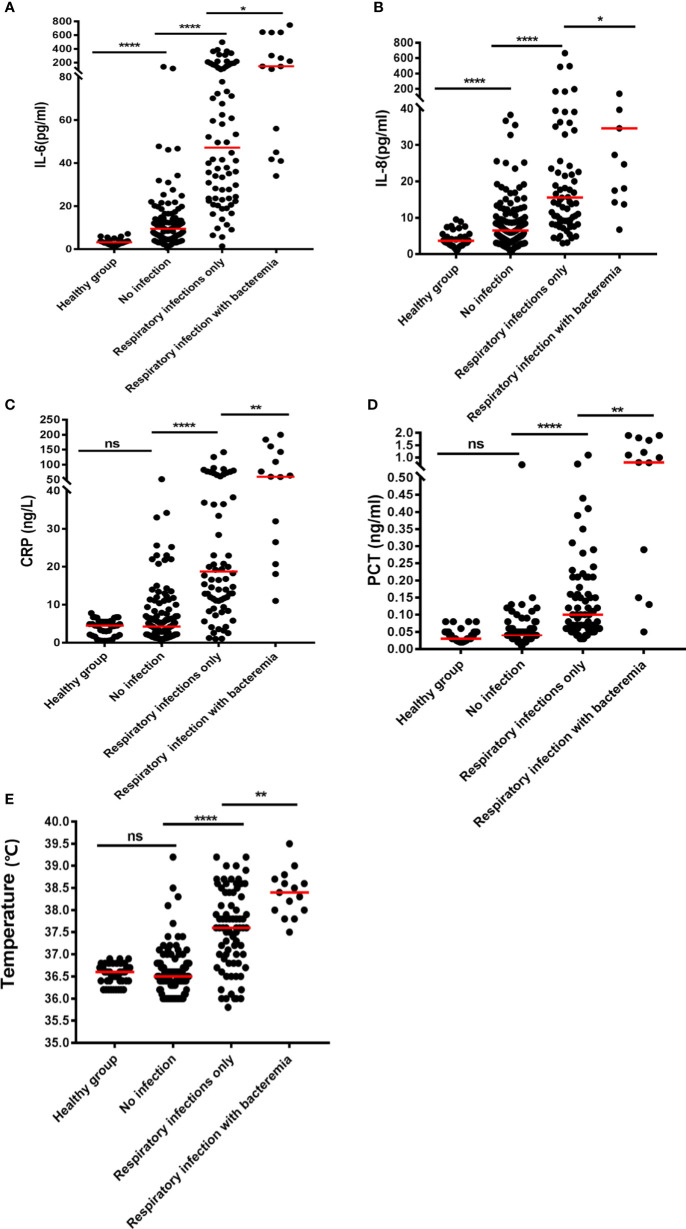
Plots of the levels of inflammatory biomarkers in NHL patients with various forms of infection: **(A)** IL-6, **(B)** IL-8, **(C)** CRP, **(D)** PCT, and **(E)** temperature. The median is shown by the horizontal lines. *P < 0.05; **P < 0.01; ****P < 0.0001; (ns) Not significant.

**Figure 2:**
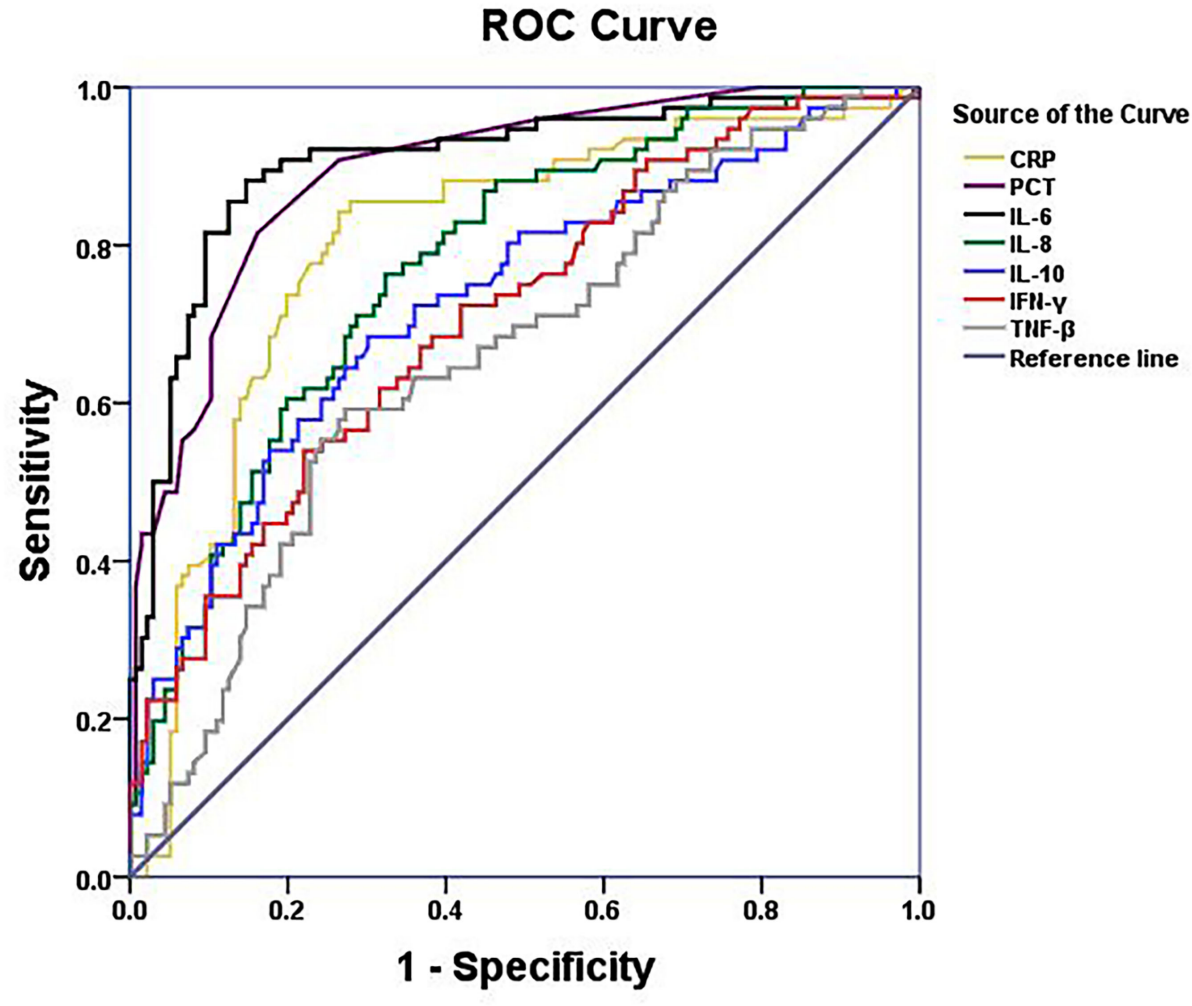
ROC curves of serum CRP,PCT, IL-6, IL-8, IL-10,IFN-γ and TNF-β levels of NHL patients with bacterial respiratory infection.

Pulmonary infection is considered to be among the most complicated hematologic malignancies to diagnose. It is common in newly diagnosed patients. Therefore, we analyzed patients with and without respiratory bacterial infection (excluding those with bacteremia). The results showed that the body temperature, CRP, PCT, IL-6, IL-8, IL-10, TNF-β, and IFN-γ levels of patients with respiratory bacterial infection were 37.6°C, 20.80mg/L, 0.10 ng/mL, 41.67 pg/mL, 15.55 pg/mL, 8.02 pg/mL, 3.82 pg/mL, and 4.76 pg/mL, respectively.While, the levels of above indicators were found to be significantly lowered in patients without infection, i.e., 36.5°C, 4.37 mg/L, 0.04 ng/mL, 9.50 pg/mL, 6.61 pg/mL, 4.52 pg/mL, 2.96 pg/mL and 2.96 pg/mL, accordingly (P < 0.05) (**Table 2** and **Figures 1A–E** and **Supplementary Figures 1A–C**). When other indicators were compared, no significant differences were observed (**Table 2**). Next, we evaluated the predictive ability of each significant indicator through the ROC curve (**Table 2** and **Figure 2**). We found that the indicators with significant AUC were body temperature (AUC = 0.82), CRP (AUC = 0.80), PCT (AUC = 0.89), IL-6 (AUC = 0.92), IL-8 (AUC = 0.78), IL-10 (AUC = 0.73), IFN-γ (AUC = 0.71) and TNF-β (AUC = 0.66), as demonstrated in **Table 3**. Cut-off values of these indicators were 37.4°C, 7.95 mg/L, 0.08 ng/mL, 18.79 pg/mL, 9.05 pg/mL, 5.74 pg/mL, 4.52 pg/mL and 3.6 pg/mL, accordingly (**Table 2**).

**Table 2:** Comparison of serum CRP, PCT and cytokine levels in NHL patients with or without bacterial respiratory infection.

Parameters	Healthy group^#^ (n=40)	No infection^#^ (n=138)	Bacterial respiratory infection only (n=76)	P-value	Cut-offvalue	AUC
Temperature (°C)	36.3 (36.2-37.0)^ns^	36.5 (36.4-36.7)	37.6 (36.8-38.1)	<0.001	37. 4	0.82
CRP (mg/L)	4.50 (1.80-5.55)^ns^	4.37 (2.19-5.64)	20.80 (12.20-66.85)	<0.001	7.95	0.80
PCT (ng/mL)	0.03 (0.03-0.05)^ns^	0.04 (0.03-0.05)	0.10 (0.06-0.21)	<0.001	0.08	0.89
IL-4 (pg/mL)	2.88 (2.08-3.97)^ns^	2.44 (1.52-3.50)	2.54 (1.65-3.95)	0.118	—	—
IL-5 (pg/mL)	1.66 (1.24-2.13)^*^	2.35 (1.84-3.16)	2.41 (1.85-3.38)	0.495	—	—
IL-6 (pg/mL)	3.22 (2.30-4.45)^****^	9.50 (5.41-13.34)	41.67 (22.31-152.78)	<0.001	18.79	0.92
IL-8 (pg/mL)	3.68 (2.42-5.54)^****^	6.61 (3.84-12.23)	15.55 (9.13-36.29)	<0.001	9.05	0.78
IL-10 (pg/mL)	4.01 (2.94-4.67)^ns^	4.52 (3.52-6.74)	8.02 (4.76-24.25)	<0.001	5.74	0.73
IL-12P70 (pg/mL)	5.54 (4.68-6.65)^***^	3.84 (3.15-4.97)	4.28 (3.60-5.46)	0.161	—	—
IL-1β (pg/mL)	1.63 (1.17-2.10)^ns^	1.84 (1.30-2.36)	1.74 (1.38-2.32)	0.859	—	—
IL-2 (pg/mL)	3.59 (2.31-5.93)^ns^	3.12 (2.29-4.30)	3.09 (1.90-4.21)	0.905	—	—
IFN-γ (pg/mL)	2.27 (1.84-3.01)^ns^	2.96 (1.98-4.41)	4.76 (2.95-9.64)	<0.001	4.52	0.71
TNF-α (pg/mL)	2.00 (1.20-2.79)^***^	3.02 (2.17-4.43)	3.18 (2.38-4.80)	0.451	—	—
TNF-β (pg/mL)	3.01 (2.72-4.00)^ns^	2.96 (1.73-3.72)	3.82 (2.62-4.39)	<0.001	3.6	0.66
IL-17A (pg/mL)	1.80 (1.03-2.84)^*^	2.52 (1.53-3.53)	2.61 (1.51-4.12)	0.476	—	—
IL-17F (pg/mL)	2.81 (1.70-3.94)^*^	3.88 (2.78-5.00)	3.18 (2.57-4.52)	0.232	—	—
IL-22 (pg/mL)	0.37 (0.11-0.77)^****^	1.04 (0.47-1.76)	1.33 (0.76-1.88)	0.057	—	—

Continuous variables were represented by median and quartile range (IQR). The optimal cut-off value was obtained by calculating the maximum Youden index (sensitivity + specificity – 1). AUC is based on the area under the ROC curve shown in **Figure 2**.

^#^Comparison of Inflammatory indicators between heatthy group and NHL patients without infection. *P < 0.05; ***P < 0.001; ****P < 0.0001; ns, not significant.

**Table 3:** Comparison on serum CRP, PCT and cytokine levels of bacterial respiratory infectious NHL patients with or without bacteremia.

Parameters	Respiratory infectious NHL patients		P-value	Cut-offvalue	AUC
	With bacteremia (n=15)	Without bacteremia (n=76)			
Temperature (°C)	38.4 (38-38.7)	37.6 (36.8-38.1)	0.001	38. 2	0.75
CRP (mg/L)	93.70 (34.70-178.36)	20.80 (12.20-66.85)	0.001	20.65	0.72
PCT (ng/ml)	0.79 (0.13-1.93)	0.10 (0.06-0.21)	0.002	0.57	0.75
IL-4 (pg/mL)	3.24 (2.35-5.84)	2.54 (1.65-3.95)	0.063	—	—
IL-5 (pg/mL)	3.19 (1.58-3.99)	2.41 (1.85-3.38)	0.744	—	—
IL-6 (pg/mL)	145.00 (33.75-640.84)	41.67 (22.31-152.78)	0.039	102.6	0.76
IL-8 (pg/mL)	34.6 (17.47-58.46)	15.55 (9.13-36.29)	0.029	24.47	0.71
IL-10 (pg/mL)	25.45 (4.92-236.42)	8.02 (4.76-24.25)	0.115	—	—
IL-12P70 (pg/mL)	4.30 (3.88-5.79)	4.28 (3.60-5.46)	0.355	—	—
IL-1β (pg/mL)	2.17 (1.39-2.92)	1.74 (1.38-2.32)	0.484	—	—
IL-2 (pg/mL)	3.55 (2.15-6.21)	3.09 (1.90-4.21)	0.158	—	—
IFN-γ (pg/mL)	6.50 (2.14-36.35)	4.76 (2.95-9.64)	0.477	—	—
TNF-α (pg/mL)	3.50 (1.97-4.97)	3.18 (2.38-4.80)	0.769	—	—
TNF-β (pg/mL)	4.38 (2.89-5.34)	3.82 (2.62-4.39)	0.246	—	—
IL-17A (pg/mL)	2.88 (1.88-6.25)	2.61 (1.51-4.12)	0.366	—	—
IL-17F (pg/mL)	4.19 (3.15-6.60)	3.18 (2.57-4.52)	0.268	—	—
IL-22 (pg/mL)	1.94 (0.51-2.88)	1.33 (0.76-1.88)	0.355	—	—

Furthermore, IL-6 (9.50 vs 3.22pg/mL, P< 0.05) and IL-8 (6.61 vs 3.68 pg/mL, P< 0.05) levels were notably greater in non-infected NHL cases than in healthy individuals (**Table 2**, **Figures 1A–E**). However, even if IL-6 and IL-8 were elevated in uninfected patients, the elevated levels were not nearly as high as in co-infected patients (**Table 2** and **Figures 1A–E**). Further comparison showed main cytokines, including IL-6 and IL-8, showed no significant difference in patients with different tumor stage (**Supplementary Table 1**). Correlation studies show that only IL-12P70 was negatively correlated with NHL stage, but the correlation was weak. No close correlation was found between other inflammatory factors and NHL stage (**Supplementary Table 2**).

### Comparison of Serum CRP, PCT, and Cytokine Levels of Bacterial Respiratory Infectious NHL Patients With or Without Bacteremia

The results in **Table 2** suggest that IL-6 is abnormally expressed in patients with a bacterial respiratory infection that can be used as a significant biomarker for the differentiation of infection. Herein, we compared patients who had a respiratory bacterial infection alone to those who had respiratory bacterial infection with bacteremia. The outcomes illustrated that the levels of body temperature, CRP, PCT, IL-6 and IL-8 in bacteremia patients were 38.4°C, 93.7 mg/L, 0.79 ng/mL, 145.00 pg/mL and 34.6 pg/mL, respectively. While the levels of these indicators in patients with respiratory bacterial infection alone were 37.6°C, 20.8 mg/L, 0.10 ng/mL, 41.67 pg/mL and 15.55 pg/mL, respectively (**Table 3** and **Figures 1A–E**, P < 0.05). Other indicators revealed no considerable discrepancies between the two groups (**Table 3**). The ROC curve was employed to further evaluate the diagnostic ability of body temperature, CRP, PCT, IL-6, and IL-8 in cases with respiratory bacterial infection and bacteremia (**Supplementary Figure 2**). In patients with respiratory bacteremia, the AUC values for body temperature, PCT, CRP, IL-6, and IL-8 were found to be 0.75, 0.75, 0.72, 0.76, and 0.71, accordingly. While their cut-off points were 38.2°C, 20.65 mg/L, 0.57 ng/ml, 102.6 pg/ml, and 24.47 pg/ml, accordingly, as indicated in **Table 3**.

## Discussion

Bacterial infection is prevalent among NHL patients associated with hematological malignancies, which often accompany the whole course of the disease and even affect the survival of patients. Among them, the pulmonary bacterial infection is difficult to diagnose in the initial stage of the infection and is further complicated by a variety of factors that can quickly proceed to severe infection. About 16% of patients’ deaths are caused by pulmonary infection (Xia et al., 2016; Periselneris and Brown, 2019). Therefore, it is critical to check for infection during the initial examination. Our obtained results revealed that IL-8 and IL-6 levels were comparatively lower in the uninfected patients than in co-infected patients. Further analysis showed that IL-6 and IL-8 are the cytokines that predict respiratory infection alone and respiratory infection with bacteremia. In particular, IL-6 is a more sensitive predictor of bacterial infection compared to PCT and CRP, and different levels of IL-6 predict different extents of infection.

IL-6 expression levels are abnormally elevated during infection. In addition, T and B lymphocyte development and differentiation are dependent on the expression of IL-6. As a pro-inflammatory cytokine, IL-6 performs a critical task in initiating antibacterial inflammation and can trigger a cascade of inflammatory mediators (Bian et al., 2017). IL-6 is produced by adipocytes, fibroblasts, keratinocytes, and endothelial cells in response to leukocyte invasion, but it is also stimulated by other cytokines, including IL-1 and TNF-α. IL-6 binds to membrane-bound IL-6R, which binds to signal transduction co-receptor glycoprotein 130 (GP130) to formulate the IL-6/IL-6Rα/gp130 complex, then signal transduction occurs through the JAK-STAT pathway (Baran et al., 2018; Naseem et al., 2018). IL-6 classical signals can induce acute responses and are regarded to have anti-inflammatory and homeostatic impacts (Rose-John, 2017). Currently, IL-6 is considered to be a multifunctional cytokine, associated with various physiological processes, such as inflammation, immune response, and hematopoiesis. The steady-state IL-6 level in healthy people is about 1-10 pg/ml (Kishimoto et al., 1995), however, its serum level is significantly elevated during infection, inflammatory disease, or cancer progression and is correlated with improper disease prognosis (Taher et al., 2018; McElvaney et al., 2021). In severe sepsis, the concentration of IL-6 can reach 100–1000 ng/mL (Jones, 2005). In this study, IL-6 levels were shown to be considerably greater in non-infected NHL cases than in healthy individuals, while IL-6 levels were significantly higher in infected NHL patients and linked with infection severity. It has been revealed that IL-6 also played a significant role in differentiating various degrees of bacterial infection in NHL patients. When IL-6 ≥ 18.79 pg/ml may suggest pulmonary bacterial infection, while IL-6 ≥ 102.6pg/ml may suggest pulmonary bacterial infection with bacteremia. This is comparable with previously published results, and IL-6 has a high value in distinguishing serious infections in clinical practices (Karan, 2002; Kitanovski et al., 2006; Xu et al., 2013). It has further been shown that the disease itself does not affect the susceptibility of IL-6 to bacterial infection in NHL patients.

Patients with respiratory bacterial infections have been shown to have abnormally expressed pro-inflammatory cytokines, including IL-6, IL-8, TNF-β, and IFN-γ. Several reported studies have shown that these cytokines can be used as predictors of diverse sorts of infection. Abnormal expression of cytokines (IL-6, IL-10, IFN-γ, and TNF) are similar in children experiencing acute lower respiratory tract infection. Furthermore, these cytokines also contribute to enterovirus infection, viral encephalitis (Fuchs et al., 2018; Xu et al., 2020), and nephritis (Mizutani et al., 2017) in children. Abnormal expression of IL-6 and IL-10 has also been found in children with acquired pneumonia (Vasconcellos et al., 2018; Nascimento-Carvalho et al., 2020). TNF-α and IFN-γ are abnormally increased in bacterial infections but seem to be more significant biomarkers in Mycobacterium tuberculosis infection. The IFN-γ/TNF-α dual release fluorescence point test is a simple and effective way to diagnose active tuberculosis (Dorhoi and Kaufmann, 2014; Kim et al., 2020; Zhang et al., 2021). IL-8 mediates its signaling through extracellular binding to two G-protein-coupled receptors (GPCRs), i.e., CXC-chemokine receptor 2 (CXCR2) and CXC-chemokine receptor 1 (CXCR1). Neutrophils can be recruited through this signal and their oxidative burst and particle release can be triggered to reduce inflammatory stimuli and boost bacterial clearance rates in inflammatory sites (such as the respiratory system) (Hartl et al., 2007; Lee et al., 2020).

IL-10, an anti-inflammatory factor, is used during infection to reduce tissue damage and immunopathology through suppressing pro-inflammatory responses induced with the aid of diverse immune cells (Engelhardt and Grimbacher, 2014; Ouyang and O’Garra, 2019). In children with hematological malignancies, greater expression levels of IL-6 and IL-10 predict infection, and their high expression indicates the severity of the infection (Xu et al., 2012; Xia et al., 2016). Our previous study also proved that IL-10 is a risk factor for early death in secondary hemophagocytic syndrome cases with severe cytokine networks (Li et al., 2021). In this study, we found that IL-10 had some predictive effect on bacterial infection and respiratory bacterial infections, but its AUC did not have a strong advantage over body temperature, CRP, and PCT (**Tables 2**, **3**, and **Figures 2A, B**). These evidences suggest that IL-10, IL-6, IL-8, TNF-β, and IFN-γ are all abnormally expressed in infection. Particularly, IL-6, IL-10, and IL-8 can serve as predictive markers of infection. In brief, the development of an inflammatory response involves the activation of several cytokines (Karan, 2002; Kitanovski et al., 2006; Tang et al., 2011; Xu et al., 2013). Monitoring the variations in cytokine levels of NHL patients is critical for guiding the prevention and treatment of the underlined infection.

We emphasized the importance of IL-6 and IL-8 in bacterial infections. Simultaneously, we suggest that patients’ cytokine levels, notably IL-6, be assessed at the time of diagnosis to help detect bacterial infection in NHL patients. Our research has following limitations: 1. Our data is a single-center study; 2. We only included patients with bacterial infections and excluded data on fungal and viral infections since fungal and viral infections were discovered in fewer cases at the time of their initial diagnosis. This will be our focus in the future.

## Conclusion

Our findings imply that an aberrant rise in many cytokines in newly diagnosed NHL cases can predict the presence of various levels of bacterial infection. IL-6, in particular, has superior sensitivity and accuracy to body temperature, PCT, and CRP. In newly diagnosed NHL patients, IL-6 ≥18.79pg/mL could indicate the existence of pulmonary bacterial infection while IL-6 ≥102.6pg/mL may suggest pulmonary bacterial infection with bacteremia. Although IL-6 level in co-infected NHL patients was significantly higher than non-infection patients and healthy people. And the benefits of IL-6 in detecting infection in NHL cases were also verified. It’s also been proposed that NHL itself may not affect the susceptibility of sensitivity to bacterial infection. Briefly, these findings may aid in the identification and distinction of lung bacterial infection and the presence of bacteremia in newly diagnosed NHL patients, as well as suggest antibiotic treatment.

## Data Availability Statement

The raw data supporting the conclusions of this article will be made available by the authors, without undue reservation.

## Ethics Statement

The studies involving human participants were reviewed and approved by The Ethics Committee of The First People’s Hospital of Yunnan Province. The patients/partici5pants provided their written informed consent to participate in this study.

## Author Contributions

All authors participated in the research and agreed to the publishing license. LZ and JPZ analyzed the data and drafted the manuscript. ZL, RZ, and XG designed the research plan, reviewed the manuscript and revised the main content. XL, FL, ZY, SZ, JLZ, and HL statistics. TY, KS, and HH checked the logic of the manuscript. All authors contributed to the article and approved the submitted version.

## Funding

The author would like to thank the patients who participated in the treatment at the First People’s Hospital and Kunming Junmai Technology Co. Ltd. for providing reagents and consumables. This work was supported by the National Natural Science Foundation of China (82060810) and Open Project of Yunnan Blood Clinical Medical Center (2019LCZXKF-XY11, 2019LCZXKF-XY03, 2019LCZXKF-XY09, 2020LCZXKF-XY07 and 2021LCZXXF-XY09).

## Conflict of Interest

The authors declare that the research was conducted in the absence of any commercial or financial relationships that could be construed as a potential conflict of interest.

## Publisher’s Note

All claims expressed in this article are solely those of the authors and do not necessarily represent those of their affiliated organizations, or those of the publisher, the editors and the reviewers. Any product that may be evaluated in this article, or claim that may be made by its manufacturer, is not guaranteed or endorsed by the publisher.
