# Supplementary figures and images for: Increased Cytokine Levels Assist in the Diagnosis of Respiratory Bacterial Infections or Concurrent Bacteremia in Patients With Non-Hodgkin’s Lymphoma
Source: Front Cell Infect Microbiol. 2022 Apr 8;12:860526. doi: 10.3389/fcimb.2022.860526 (PMC9024136; doi:10.3389/fcimb.2022.860526)

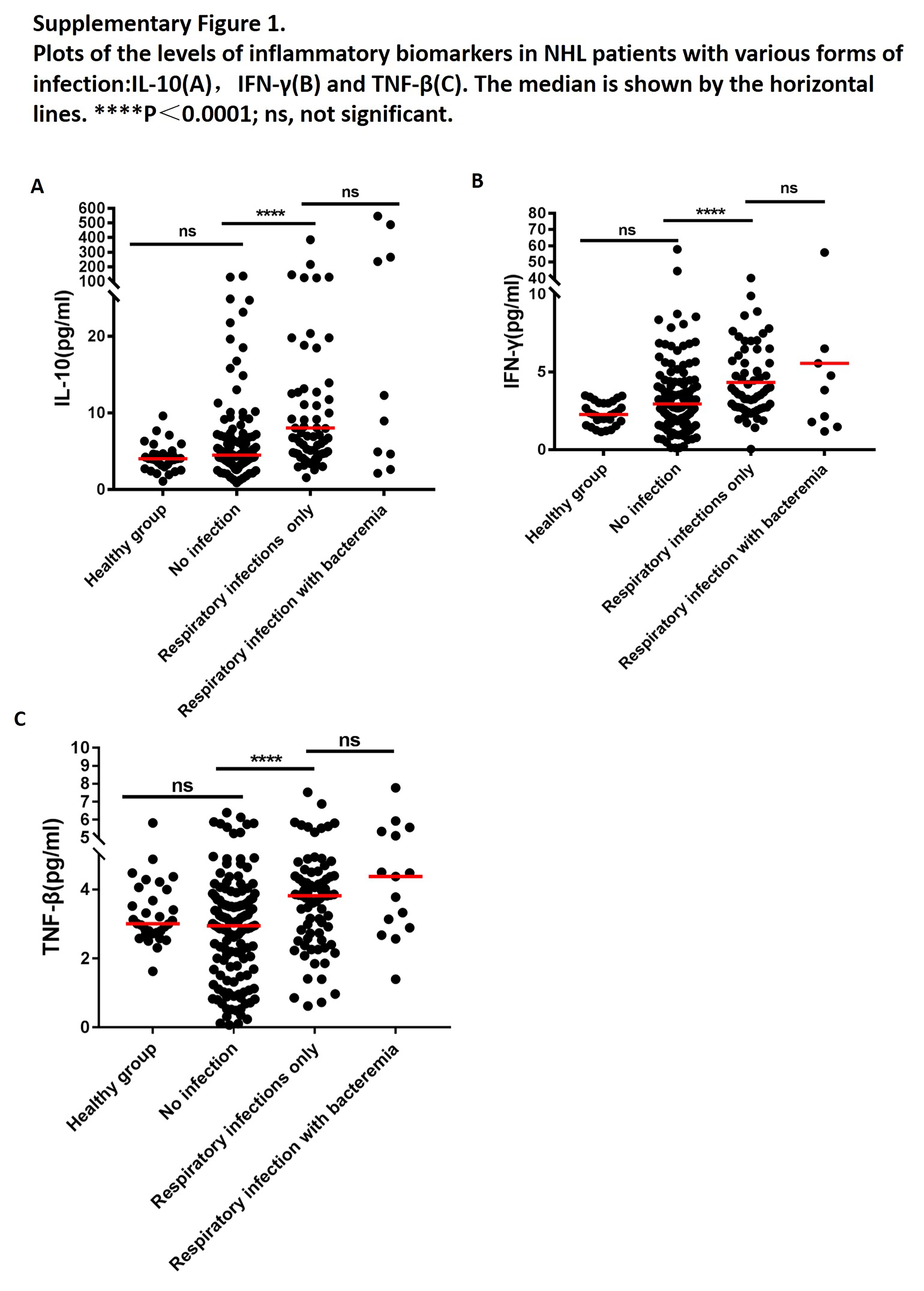

Supplement: Supplementary file 1 [file Image_1.tif]

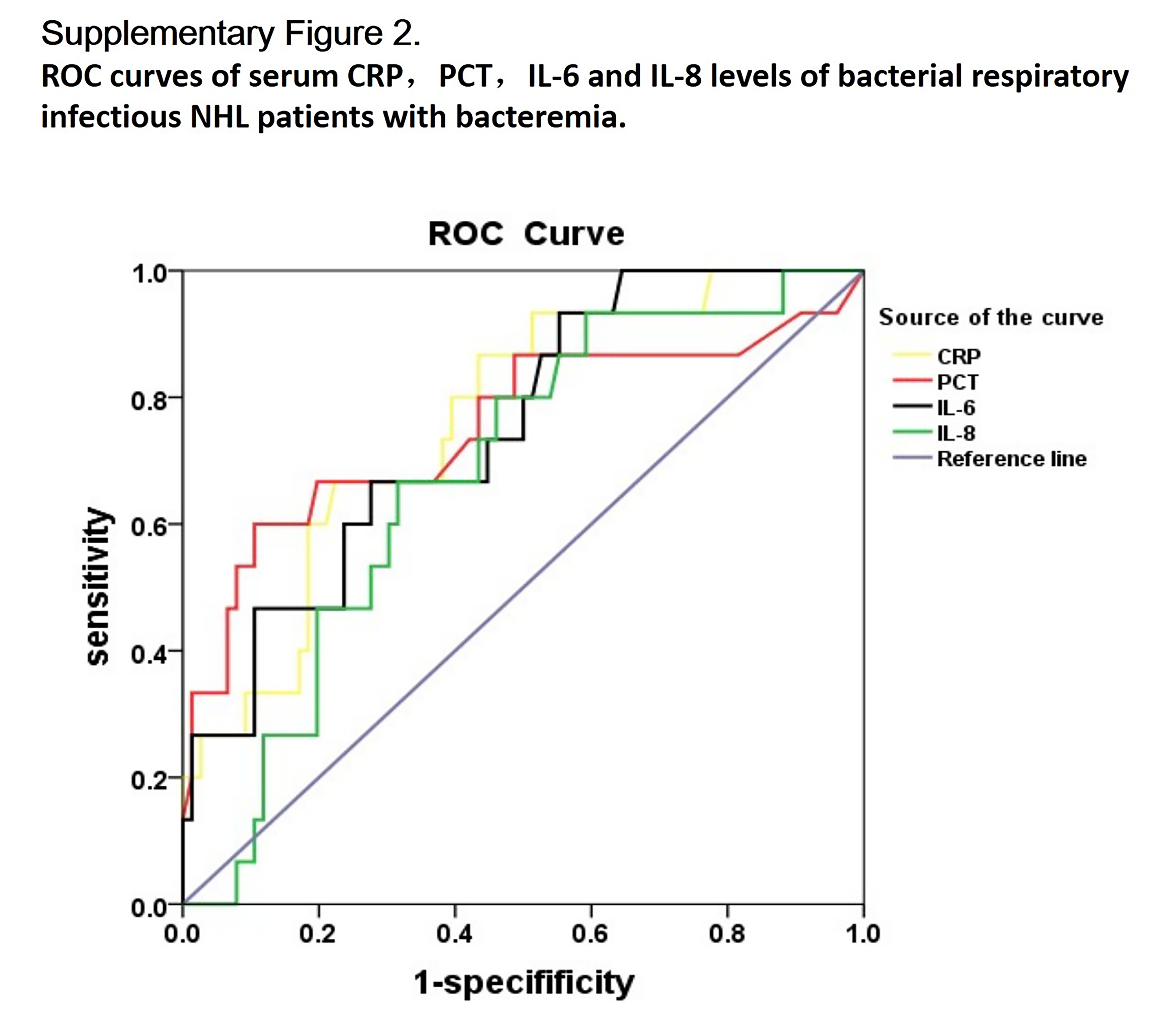

Supplement: Supplementary file 2 [file Image_2.tif]
