# Supplementary material for: Increased Cytokine Levels Assist in the Diagnosis of Respiratory Bacterial Infections or Concurrent Bacteremia in Patients With Non-Hodgkin’s Lymphoma
Source: Front Cell Infect Microbiol. 2022 Apr 8;12:860526. doi: 10.3389/fcimb.2022.860526 (PMC9024136; doi:10.3389/fcimb.2022.860526)
Supplement: Supplementary file 3 [file DataSheet_1.docx]

**Supplementary Table 1.**

Comparison of levels of inflammatory factors in non-infected NHL patients with different tumor stage.

|  | Tumor stage | |  |
| --- | --- | --- | --- |
|  | Ⅰ-Ⅱ(n=34) | Ⅲ-Ⅳ(n=104) | P-value |
| CRP(mg/L) | 4.52(1.69-7.67) | 4.28(2.20-11.00) | ns |
| PCT(ng/mL) | 0.03(0.02-0.05) | 0.04(0.03-0.05) | ns |
| IL-4(pg/mL) | 2.67(1.47-3.32) | 2.43(1.52-3.64) | ns |
| IL-5(pg/mL) | 2.40(1.81-3.21) | 2.33(1.84-3.20) | ns |
| IL-6(pg/mL) | 8.49(5.42-15.26) | 9.63(5.36-13.01) | ns |
| IL-8(pg/mL) | 7.53(4.31-10.21) | 6.32(3.74-12.47) | ns |
| IL-10(pg/mL) | 4.49(3.26-6.67) | 4.59(3.71-6.80) | ns |
| IL-12P70(pg/mL) | 4.49(3.43-5.84) | 3.73(3.11-4.66) | 0.048 |
| IL-1β(pg/mL) | 1.74(1.12-2.88) | 1.85(1.50-2.36) | ns |
| IL-2(pg/mL) | 3.48(2.56-4.88) | 3.02(2.21-3.73) | ns |
| IFN-γ(pg/mL) | 3.32(2.06-6.45) | 2.88(1.88-4.35) | ns |
| TNF-α(pg/mL) | 2.89(2.04-4.81) | 3.15(2.20-4.19) | ns |
| TNF-β(pg/mL) | 3.03(1.33-3.56) | 2.88(1.79-3.88) | ns |
| IL-17A(pg/mL) | 2.65(1.49-3.68) | 2.50(1.54-3.46) | ns |
| IL-17F(pg/mL) | 3.96(2.61-6.07) | 3.83(2.82-4.84) | ns |
| IL-22(pg/mL) | 1.21(0.73-2.50) | 0.97(0.43-1.75) | ns |

**Supplementary Table 2.**

The relationship between the tumor stage and inflammatory factor levels in the NHL patients.

| Variables | P-value | Spearman Rank |
| --- | --- | --- |
| CRP(mg/L) | ns | 0.048 |
| PCT(ng/mL) | ns | 0.047 |
| IL-4(pg/mL) | ns | 0.005 |
| IL-5(pg/mL) | ns | -0.006 |

| IL-6(pg/mL) | ns | -0.001 |
| --- | --- | --- |
| IL-8(pg/mL) | ns | 0.002 |
| IL-10(pg/mL) | ns | 0.085 |
| IL-12P70(pg/mL) | 0.048 | -0.169^*^ |
| IL-1β(pg/mL) | ns | 0.051 |
| IL-2(pg/mL) | ns | -0.132 |
| IFN-γ(pg/mL) | ns | -0.125 |
| TNF-α(pg/mL) | ns | 0.010 |
| TNF-β(pg/mL) | ns | 0.001 |
| IL-17A(pg/mL) | ns | -0.015 |
| IL-17F(pg/mL) | ns | -0.019 |
| IL-22(pg/mL) | ns | -0.136 |
